# Effect of Process Parameters on Plasma-Enhanced Solvolysis of CFRPs

**DOI:** 10.3390/ma18225081

**Published:** 2025-11-08

**Authors:** Dimitrios Marinis, Ilektra Tourkantoni, Ergina Farsari, Eleftherios Amanatides, Konstantinos Tserpes

**Affiliations:** 1 Department of Chemical Engineering, University of Patras, GR26504 Patras, Greece; marinis@chemeng.upatras.gr (D.M.); efarsari@chemeng.upatras.gr (E.F.); 2 Department of Mechanical Engineering and Aeronautics, University of Patras, GR26504 Patras, Greece; ilektra.tourkantoni@ac.upatras.gr (I.T.); kitserpes@upatras.gr (K.T.)

**Keywords:** recycling, CFRP, nitric acid, carbon fibers, plasma liquids, solvolysis

## Abstract

The current study investigates plasma-assisted chemical recycling as an innovative approach to recover valuable carbon fibers from composite waste while minimizing environmental impact. Nitrogen and argon plasma-in-bubbles are employed in a concentrated nitric acid solution, thus enhancing conventional nitric acid solvolysis with plasma chemistry. A systematic process framework is presented, revealing key operational stages, including composite pretreatment, composite solvolysis, carbon fiber recovery/characterization, NO_x_ recovery, nitric acid circulation, and byproduct management, demonstrating their role in the overall process efficiency and environmental impact. Moreover, the research examined different processing conditions, including plasma power, acid concentration, and reactor design, while comparing open-air systems to systems equipped with single-stage or two-stage wet scrubbers for NO_x_ recovery. Remarkably, recycled fibers from plasma-assisted solvolysis demonstrated preserved or even slightly enhanced mechanical properties compared to those of the virgin fibers. Recycled carbon fibers originating from the operation at 1200 W and 12 M HNO_3_ demonstrated the best mechanical properties with 3138.92 MPa tensile strength and 307.02 GPa Young’s modulus. However, the parametric analysis revealed that operating the plasma reactor at 1200 W and 14 M, equipped with a two-stage scrubber, achieved optimal environmental performance.

## 1. Introduction

Carbon fiber-reinforced polymers (CFRPs) continue to have a strong impact on the growth and development of many weight-dependent industries, such as wind energy, aeronautics, and automotive industries, due to their excellent physicochemical and mechanical properties. These properties are attributed to the synergy between the low density and high strength of CFRPs, offering a lightweight, robust structure that is ideal for advanced applications and extreme conditions [1,2]. However, the increased usage of carbon fibers has raised awareness of waste management options. Every year, tons of end-of-life composites accumulate from numerous sectors, triggering environmental concerns due to the substantial waste they generate. Thus, environmentally friendly recycling of composite waste is essential. Since traditional disposal methods, like landfilling and burning, are restricted due to their environmental and resource impacts, alternative processing methods are needed [3,4,5].

The most mature retrieval methods are classified into three categories, i.e., mechanical, thermal, and chemical recycling [6,7,8,9,10,11,12]. Mechanical recycling involves shredding or milling the solid waste into flakes, short fibers, and resin-rich powders that are typically used as fillers or reinforcements to fix or improve other materials. However, while mechanical recycling is technologically mature and economically attractive for large, low-performance waste streams, it cannot yet deliver high-quality fibers for demanding aerospace or automotive parts [13,14,15,16].

On the contrary, thermal and chemical methods aim to reclaim the fibers by removing the polymeric matrix of the composite. Thermal recycling focuses on pyrolysis-based techniques, where the composite waste is heated at temperatures between 400 and 700 °C in an inert atmosphere, effectively decomposing the polymer matrix and yielding clean fibers while utilizing byproducts like gases and resin oils for energy, and thus supporting the overall process of sustainability. However, thermal processes have high energy requirements while inducing gas emissions such as CO_2_ and CO [17,18,19].

In response, chemical recycling, or solvolysis, has emerged as a promising alternative. This technique uses targeted chemical reactions (e.g., hydrolysis, alcoholysis, glycolysis, acid/base solvolysis, or reversible-epoxy systems), selectively cleaving critical chemical bonds of the crosslinked network [20,21,22,23,24,25,26,27]. The selective bond cleavage leads to the dissolution of the polymer matrix and the retrieval of clean and nearly intact fibers, while recovering valuable monomers is, in some cases, also possible. Overall, solvolysis, with a suitable solvent and the right conditions, can efficiently liquefy the resin matrix under mild conditions, minimizing fiber damage. These attributes make solvolysis one of the most promising recycling technologies for CFRPs, offering a possible sustainable solution to the growing waste management challenges [11,28].

Typical sub-routes of chemical recycling include hydrolysis, alcoholysis, and acidolysis. In hydrolysis, water can cleave ester and ether bonds in polymeric matrices, often under acidic or alkaline conditions, operating in subcritical (180–374 °C, 1–25 MPa) or supercritical (>374 °C, >22 MPa) conditions. Typical processes achieve >95% resin removal, with recovered fibers retaining 85–95% of the virgin tensile strength. However, high-pressure autoclaves drive up capital costs and safety requirements, while energy demands (12–19 MJ kg^−1^ fiber) remain substantial compared to other routes [29,30,31]. In alcoholysis, monohydric alcohols (e.g., methanol and ethanol) in liquid or supercritical form are employed to transesterify polyester matrices into esters and glycol. This method can yield fibers with a tensile strength retention of 88–97% and monomers directly capable of reproduction, but its disadvantages involve the high energy demand and the need for explosion-proof equipment. Glycolysis, a specific subtype of alcoholysis, uses di-hydric alcohols (commonly, ethylene glycol or PEG), plus a transesterification catalyst at 180–240 °C, and near-atmospheric pressure to cleave ester linkages. This route achieves >95% resin reclamation, recovers fibers with 90–98% of their original mechanical properties, and requires simple glass reactors with lower energy input compared to the other chemical routes, but with high decomposition time [10,22,31,32,33].

Acid solvolysis, also called wet oxidation, uses concentrated acidic solutions, such as nitric acid, acetic acid, and hydrogen peroxide, to depolymerize resin matrices of CFRPs, releasing clean, intact fibers with tensile strength retention > 85–95%. Wet oxidation methods are carried out at low temperatures (80–150 °C) and atmospheric pressure, and they have the advantage of decomposing unknown types of CFRP scrap. The main disadvantage of this family of techniques is the generation of acid waste streams that require treatment [34,35,36].

Nitric acid is recognized as the most effective oxidant in terms of decomposition time and solvent reusability, ensuring recovered fibers with up to 98% of their original strength. Nitric acid solvolysis offers a relatively mild, atmospheric-pressure route at moderate temperatures (80–120 °C). During this method, acid protonates and oxidatively cleaves C–O and C–N bonds of the crosslink network, dissolving resin into soluble oligomers. Consequently, the post-treatment mixture consists of residual nitric acid and nitrated resin oligomers, whose recovery is quite challenging. The recovered clean carbon fibers also acquire polar nitro/amine surface groups that can enhance adhesion in remanufactured parts. The process requires corrosion-resistant reactors and downstream neutralization to handle NO_x_ emissions but avoids high pressures, inert atmospheres, and energy-intensive heating, making it a straightforward, scalable option for closed-loop recycling [21,34,37,38,39,40,41].

Recent works show that plasma-assisted nitric acid solvolysis combines the chemical action of HNO_3_ with non-thermal plasma generated directly in bubbles to accelerate the decomposition time and enhance fiber recovery. Specifically, plasma inside liquids generates highly reactive and short-lived species that are typically inaccessible under atmospheric pressure and low temperature conditions. In a plasma reactor, nitrogen or/and argon plasma is ignited into a concentrated HNO_3_ solution through an electrical discharge. Thus, producing highly reactive species into the solution (e.g., •OH, •OH_2_, •O, •NO_2_) that accelerate the breakage of the C–O and C–N bonds far more rapidly than nitric acid alone. This synergistic process can dissolve the matrix in ~4–12 h (vs. >8–24 h for conventional HNO_3_ solvolysis), recovering fibers with >85% tensile strength retention and introducing nitro/amine surface functional groups that boost interfacial shear strength [32,42,43,44,45,46,47,48,49]. The aim of the current work is the development of a complete set of processes for the retrieval of CFs through plasma-enhanced nitric acid solvolysis. The group of processes includes composite pretreatment, solvolysis, CFs cleaning, and treatment of gas and liquid wastes. While plasma-assisted nitric acid solvolysis shows promise for CFRP recycling, critical gaps remain regarding systematic process optimization, environmental impact assessment, and integrated waste management. Previous studies have not investigated how reactor design, acid concentration, and plasma power simultaneously affect treatment times, mechanical properties, chemical consumption, and NO_x_ emissions [32,42,45,46,47]. This work aims to close these gaps by systematically evaluating plasma-assisted solvolysis on various configurations, with a particular focus on emissions and waste minimization strategies. The study provides a comprehensive assessment of how the main operational parameters affect CF retrieval rates, CF mechanical properties, HNO_3_ losses, and gas emissions. By identifying optimal processing windows, this work provides basic guidelines for further optimization towards scale-up.

## 2. Materials and Methods

**Figure 1:**
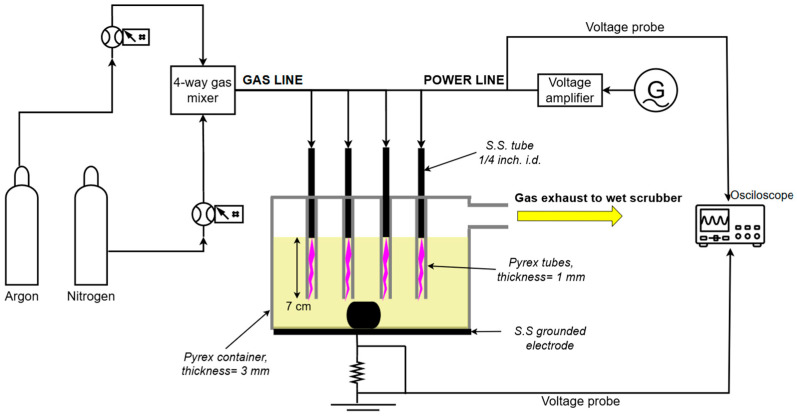
Plasma reactor setup.

Figure 1 depicts the plasma reactor setup for the plasma-assisted solvolysis of the carbon fiber-reinforced polymers (CFRPs). The reactor consists of a 2 L glass container where concentrated (40–65% wt/wt, Lach-ner, Neratovice, Czech Republic) HNO_3_ can be placed with a maximum CFRP capacity of 1 kg. The glass container is placed on the top of a stainless-steel plate that is connected to the ground. Four powered electrodes that are stainless-steel tubes are also placed inside the reactor and are adjusted to the surface of the solution. The electrodes are also surrounded externally by glass tubes that are immersed 7 cm into the solution, allowing the flowing gas (N_2_ > 99.9% and Ar > 99.9%, Novogas, Hamilton, ON, Canada) to produce bubbles. The flow ratio is maintained at $\frac{Ar}{N_{2}}=4$, utilizing two flow controllers (Hi-Tec EL-FLOW Select Mass flow controller F-201C-FAC-8-V, Bronhorst, Maharashtra, India, and MF1 Compact General-Purpose Mass flow controller, MKS Instruments, Andover, MA, USA). A 30 KHz high-frequency generator (IGBT143, Martignoni Elettrotecnica, Milano, Italy) is used for plasma ignition through a voltage amplifier (IGBT163, Martignoni Elettrotecnica).

During operation, plasma is generated within the nitric acid solution through high-voltage discharge, producing reactive species that disperse throughout the reactor to facilitate resin matrix degradation. The applied high voltage is distributed uniformly across four plasma heads that are parallel to the ground connection and recorded with a high-voltage passive probe (1000:1, P6015A, Tektronix, Beaverton, OR, USA). Current flow through the system is determined by measuring the voltage drop across a 6.8 Ω precision resistor (HS300 6R8 J ±5%, Arcol, Cornwall, UK) connected between the stainless-steel bottom electrode and ground. These voltage and current measurements enable real-time calculation of the discharge power, which, for the present set of experiments, varies from 250 to 530 W based on the generator’s input settings.

For the solvolysis process, custom-manufactured tubes provided by B&T Composites (Florina, Greece) were used. The composite materials were produced by using the filament-winding method. EPIKOTE™ Resin 828 and EPIKURE™ Curing Agent 866 (anhydrite agent) (Westlake Epoxy, Houston, TX, USA) were used for the matrix, and each sample contained 24 m of continuous 24 K carbon fibers (CFs) (Tenax^®^-E STS40 E23 24 K 1600tex, Tenax Corporation, Baltimore, MA, USA). Each tube specimen had an approximate volume of 2 cm^3^ and mass of 39 ± 2 g, with a nominal fiber mass content of 64 ± 3%. The volume of the HNO_3_ solution was set equal to 1.2 L while the flow rates of N_2_ and Ar were set equal to 0.12 L·min^−1^ and 0.48 L·min^−1^, respectively.

**Figure 2:**
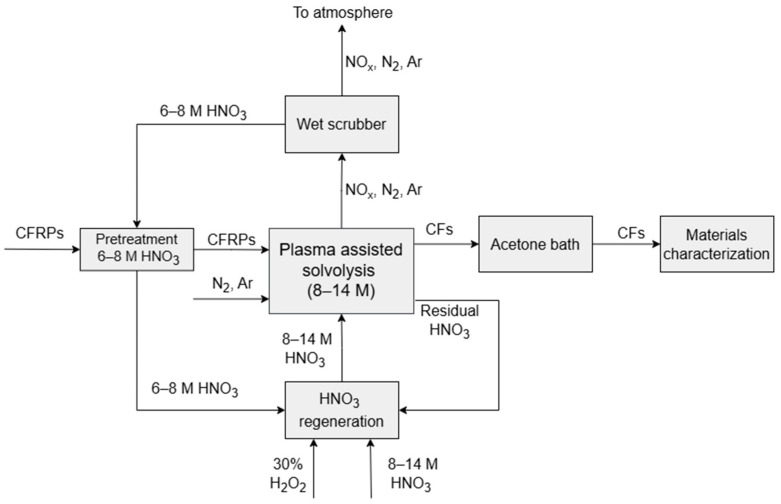
Flow diagram of the process.

Concerning the complete layout of the carbon fiber recycling process, several additional steps are required to ensure both the quality of the final product and the waste treatment of the overall process. Figure 2 illustrates the entire flow diagram, where techniques such as CFRP pretreatment, solvent regeneration, flue gas scrubbing, and carbon fiber cleaning are carried out in conjunction with the plasma process. Specifically, the CFRPs are pretreated in a 6–8 M HNO_3_ solution for several weeks before entering the plasma reactor, where they are treated until there is a complete degradation of the polymeric matrix. During the treatment, the emitted gas mixture is led into a wet scrubbing system, consisting of a low-concentration HNO_3_ and H_2_O_2_ solution. The purpose of the wet scrubber is to partially remove the NO_x_ compounds from the exhaust stream of the process through liquid-phase absorption. It is worth noting that as the NO_x_ gas was absorbed, the scrubbing liquid was enriched with NO_3_^−^, which in turn allows its use either as the composite’s pretreatment liquid or as the process solution. After the end of the dissolution process, the residual solvent from the plasma reactor undergoes a regeneration process and is reused multiple times. Specifically, residual HNO_3_ solution is mixed with fresh HNO_3_ up to the volume of 1.2 L H_2_O_2_, or with the scrubbing liquid until it reaches the desired volume and concentration for the next plasma operation cycle. Finally, the carbon fibers are mechanically collected and washed with acetone and water. The recovered CFs are characterized by means of SEM (JEOL 6300, JEOL, Zhubei City, Taiwan) and EDS (Link ISIS 300, Oxford Instruments, High Wycombe, UK) on 20 kV accelerating voltage. Meanwhile, their tensile properties are measured using a Miniature Materials Tester (Minimat 2000) (Rheometric Scientific, Inc., Piscataway, NJ, USA) according to ASTM C1557-14 [50]. For each condition, thirty single-fiber specimens were tested. Only fibers that fractured within the gauge length and passed outlier rejection using the interquartile range (IQR) criterion were considered valid, resulting in approximately twenty-five effective specimens per condition.

As for the NO_x_ absorbance process, two different scrubbing systems were tested: a single-scrubber system and a two-scrubber system. Concerning the single-stage configuration, it consisted of a 3 L mixture solution of 0.06 M H_2_O_2_ and 0.01 M HNO_3_ in a ~5 L polypropylene rectangular container with external dimensions of 20 cm × 14 cm × 23.5 cm (length × width × height). The two-stage wet scrubber involved two 3 L stainless-steel cylindrical tubes connected in series, each containing 1.5 L of wet scrubbing solution (0.06 M H_2_O_2_–0.01 M HNO_3_). Each tube had an outer diameter of 10.5 cm, a height of 36.5 cm, and a 0.1 cm wall thickness. In both configurations, flue gas from the plasma reactor was released into the solution in the form of bubbles through a ¼″ I.D stainless-steel tube, whose outlet was positioned 0.5 cm above the bottom of each vessel. In both cases, the acidity of the scrubbing liquids was systematically determined through acid–base titration using 1 M NaOH as titrant.

## 3. Results and Discussion

To examine the process performance, several experiments were performed over a range of HNO_3_ concentrations (8–14 M) and generator power inputs (1200–1700 W) in two system configurations: (a) an open-air system with ventilation and (b) the plasma reactor connected to the single scrubber. Experimental investigation of plasma-assisted recycling of one CFRP specimen in each case demonstrates significant performance variations depending on reactor configuration, acid concentration, and generator power input.

**Figure 3:**
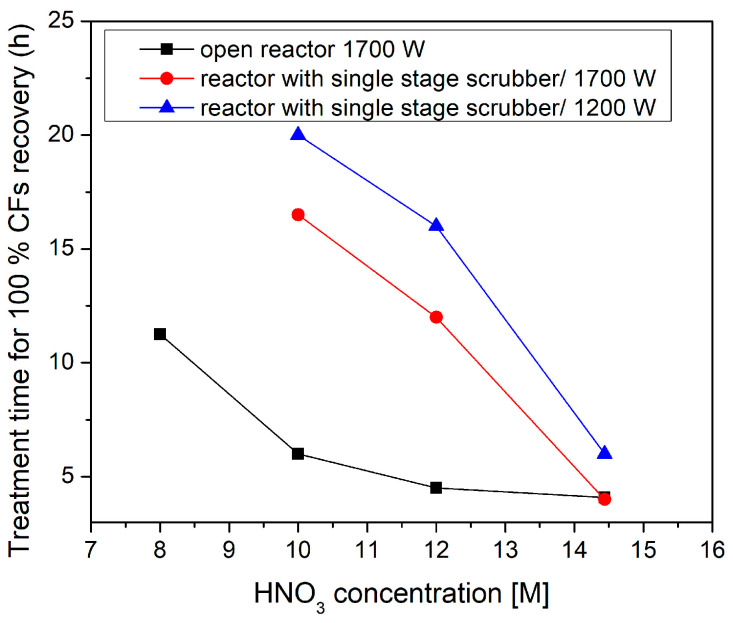
Treatment time that is required for 100% degradation of one carbon fiber-reinforced polymer (CFRP) specimen (39 ± 2 g) as a function of the solvent concentration (M) for the open reactor configuration at 1700 W power input and a single scrubber-equipped reactor at 1200 and 1700 W power input.

Figure 3 illustrates the time needed for complete fiber detachment of a single specimen as a function of HNO_3_ concentration for both configurations. The open reactor configuration consistently demonstrates superior performance for concentrations between 8 and 12 M compared to the scrubber-equipped reactor, in the same power input (1700 W). Specifically, in the open-air reactor, increasing HNO_3_ concentration from 8 to 14 M accelerates degradation, with higher acid molarity providing a greater population of reactive nitric and radical species to attack the polymer matrix. Consequently, treatment time decreases progressively, from 11.5 h at 8 M to 4.5 h at 12 M, before plateauing at 4 h at 14 M. The open reactor’s unrestricted venting allows gaseous byproducts (CO_2_, NO_2_, and H_2_O) to escape, preventing back pressure that would slow the ongoing chemical reactions. This continuous removal of byproducts prevents the establishment of chemical equilibrium states that would slow the degradation. Additionally, unrestricted gas flow due to ventilation results in enhanced bubble dynamics at the plasma–liquid interface, promoting superior mass transfer rates through improved surface renewal and mixing [51].

On the other hand, for the reactor equipped with a single-stage wet scrubber and a 1700 W power input, a stronger concentration dependency is revealed. For an 8 M HNO_3_ concentration, 16.5 h are required, and even at 12 M, treatment time remains elevated at 12 h, indicating mass-transfer limitations due to restricted gas flow pathways. The accumulation of nitrogen oxides, water vapor, and other volatile reaction byproducts creates diffusion barriers that reduce the effective concentration gradient on the solvolysis process [52,53,54]. Only at 14 M, the wet scrubber configuration overcomes its diffusion barriers, where solvolysis becomes so rapid that the mass-transfer resistances no longer impose significant limitations, thus matching the open reactor’s ~4 h treatment time at 1700 W. Therefore, the recovery rate is favored by the increase in acid concentration for both configurations. In the open reactor, reasonable dissolution times can be achieved for HNO_3_ concentrations higher than 10 M. In the single-scrubber reactor system, HNO_3_ solutions exceeding 12 M are required to overcome the mass-transfer limitations and achieve treatment times comparable to the open reactor.

**Figure 4:**
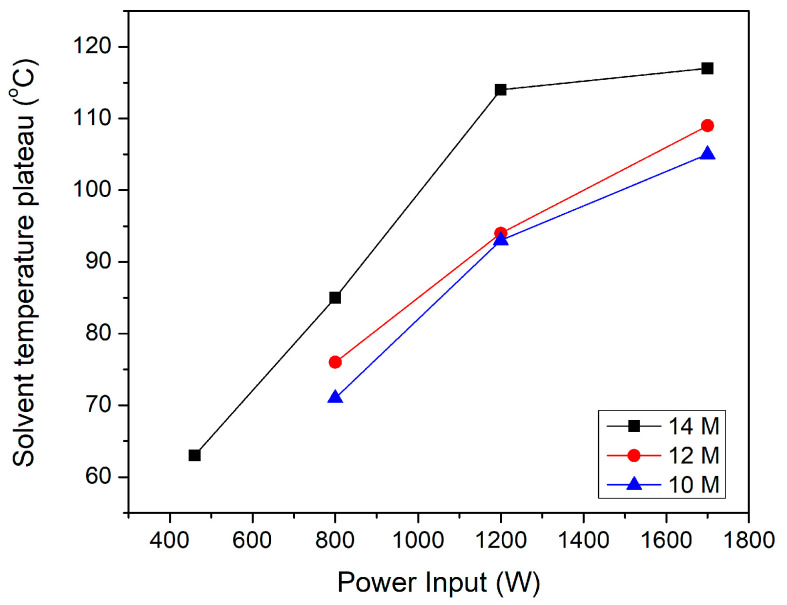
Solvent plateau temperature as a function of power input for different HNO_3_ concentrations.

The effect of the power input is also illustrated in Figure 3 for the scrubber-equipped reactor. As the generator power increases from 1200 W to 1700 W, a significant decrease in treatment time is observed regardless of the HNO_3_ concentration, indicating the combined effect of plasma-induced heating and plasma chemistry. Figure 4 presents the solvent temperature as a function of power input for different HNO_3_ concentrations. The increase in plasma power induces higher heat transfer through more energetic collisions of plasma species with solvent molecules, thus leading to elevated solvent temperature. In turn, elevated temperatures improve the solubility of intermediate resin products in the nitric acid medium, preventing the formation of insoluble residues that could slow down degradation [55,56].

However, enhanced solubility at higher temperatures is not the only reason for the increase in retrieval rates with plasma power. For instance, at 14 M concentration, the solution temperature is almost the same at 1200 W and 1700 W (Figure 4), but a much lower treatment time was detected for the input power of 1700 W (Figure 3). This temperature-independent performance difference between 1700 W and 1200 W power input demonstrates that plasma power contributes significantly to the efficiency of the process. Higher discharge power intensifies electric fields at the plasma–liquid interface, generating more reactive species (•OH, •OH_2_, •O, •NO_2_) that effectively attack polymer chains. Additionally, at 1700 W, stronger electric fields create more vigorous bubble dynamics, enhancing mixing and mass transfer at the plasma–liquid interface. The elevated plasma power at 1700 W also creates stronger electrochemical activation of the nitric acid, producing higher concentrations of oxidizing intermediates that attack the polymer matrix [51,53,57]. Overall, our findings indicate that plasma-assisted solvolysis benefits from both plasma chemistry and plasma-induced heating.

**Figure 5:**
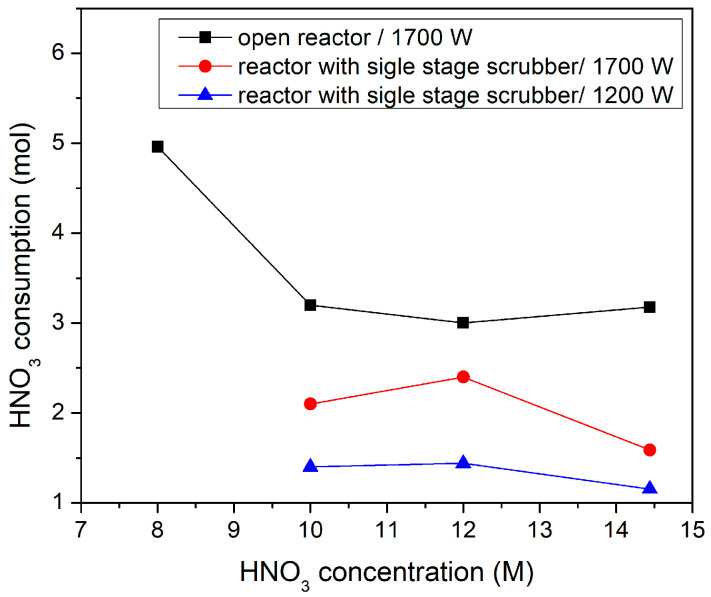
HNO_3_ consumption (mol) as a function of HNO_3_ concentration (M) for the degradation of one carbon fiber-reinforced polymer (CFRP) specimen (39 ± 2 g) via plasma-assisted solvolysis.

Another important parameter of significant impact on the process sustainability is the solvent losses due to evaporation (high flow rates and heating). Figure 5 illustrates the moles of HNO_3_ as a function of the solvent concentration for the complete degradation of one specimen and for both reactor configurations. The observed HNO_3_ losses outline that the open reactor at 1700 W exhibits the highest consumption due to evaporation, primarily because ventilation continuously strips acid vapor from the solution. At lower acid concentrations, the HNO_3_–water mixture has a lower boiling point, increasing vaporization. In the open-air system, this vapor escapes freely since there is minimal containment of evaporated HNO_3_. Therefore, the losses approach 5.0 mol at 8 M, while at higher concentrations, the losses are slightly above 3 mol. In contrast, the scrubber-equipped reactor limits vapor escape, reducing the losses to 2.1–2.4 mol at 10–12 M and further down to 1.6 mol at 14 M when operated at 1700 W. When even lower power is employed (1200 W), the operation yields minimal evaporation of 1.1–1.4 mol across all concentrations due to (a) reduced solvent temperatures (Figure 4), (b) restricted gas release, and (c) milder bubble dynamics due to less intensive plasma. Overall, the increase in plasma power favored the CF retrieval rates, but at the same time enhanced HNO_3_ losses and NO_x_ emissions due to plasma-induced heating and elevated evaporation rate. Thus, a power tuning to intermediate values is necessary to preserve relatively high CFs retrieval rates without unnecessary HNO_3_ losses. For the specific configuration, the optimum power was 1200 W.

Thus, based on minimal HNO_3_ evaporation and NO_x_ emissions, the scrubber-equipped system operating at 1200 W was selected for detailed wet scrubber performance analysis. For the wet scrubber evaluation, the solvent losses during plasma treatment, the acidity, and the volume of the scrubbing liquid were considered, and the amount of nitrogen oxide species (NO_x_) escaping the scrubbing system was calculated. Since high temperature promotes N_2_O_4_ decomposition to NO_2_, which is also observed by the reddish-brown color of the plasma-treated HNO_3_ solutions, we can assume that the plasma reactor vapors consist mostly of HNO_3_ aerosols due to solvent turbulence and NO_2_ as a result of HNO_3_ heating. Thus, the molar amount of NO_x_ that escapes the system will be equal to the HNO_3_ losses minus the amount of NO_x_ that was absorbed or condensed in the scrubber:

$$E_{NOX}=V_{R}\cdot C_{R}{-V}_{WS}\cdot C_{WS}$$

where *V_R_* is the volume of the solvent that was lost during plasma treatment, *C_R_* is the molar concentration of the solvolysis solution, *V_WS_* is the volume of the scrubbing liquid, and $C_{WS}$ is the molar NO_x_ concentration of the scrubber as determined by acid–base titration.

**Figure 6:**
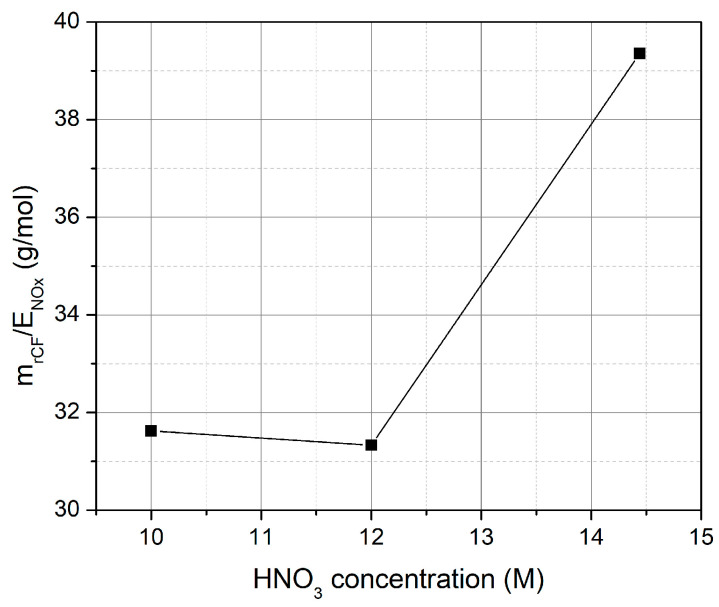
Ratio between the recovered carbon fibers’ mass (m_rCFs_) and the NO_x_ emissions (E_NOX_) as a function of HNO_3_ concentration (M) for recycling of one carbon fiber-reinforced polymer (CFRP) specimen using the reactor with a single-stage scrubber at 1200 W.

Figure 6 presents the ratio of recovered carbon fibers’ mass to the moles of the emitted NO_x_ $\left(\frac{m_{rCFs}}{E_{NOX}}\right)$ as a function of nitric acid concentration, revealing critical insights into process environmental impact. The data demonstrates a significant increase in the $\frac{m_{rCFs}}{E_{NOX}}$ ratio from approximately 31 g/mol at 10 M to ~40 g/mol at 14 M HNO_3_, indicating substantially improved environmental performance at higher acid concentrations. This enhancement reflects the synergistic effects of reduced treatment times (Figure 3) and lower absolute NO_x_ generation (Figure 5), despite the poor performance of the single-stage wet scrubber (48% NO_x_ recovery). The superior performance at 14 M concentration can be attributed to the rapid solvolysis that minimizes the total operational time and, consequently, reduces total NO_x_ emissions. The elevated $\frac{m_{rCFs}}{E_{NOX}}$ ratio at 14 M reflects the process reaching optimal chemical efficiency, enabling complete composite breakdown with minimal byproduct generation. Based on these findings, the optimal HNO_3_ concentration is revealed at 14 M HNO_3_, which achieves the highest mass of recovered carbon fibers to NO_x_ emissions ratio, while retaining similar treatment times compared to the open-air systems (4–6 h). The shorter treatment times and the lower evaporation rates were identified as the main reasons for the better performance of 14 M solutions compared to lower concentrations. However, the single-stage wet scrubber design is limited to 48% NO_x_ recovery efficiency, representing a significant bottleneck for process sustainability. Thus, the implementation of the two-stage scrubbing system was examined. Table 1 summarizes the performance of the single-stage and two-stage scrubbing systems.

The two-stage wet scrubber design aims to maximize NO_x_ capture and further improve the overall environmental impact of the process compared to the initial single-stage system. Specifically, under 14 M HNO_3_ at 1200 W power input, the two-stage scrubber achieves 1.58 mol HNO_3_ consumption, slightly higher than the single-stage scrubber (1.15 mol), which can be attributed to the increased back pressure. Moreover, the two-stage system reaches 82.7% NO_x_ recovery, nearly doubling the efficiency compared to the initial single-stage design (48%), while maintaining a 6 h treatment time. The improved capture efficiency increases the recovered carbon fibers to NO_x_ emissions ratio $\left(\frac{m_{rCFs}}{E_{NOX}}\right)$ from 39 to 96 g/mol, significantly reducing environmental impact and downstream NO_x_ treatment needs. By dividing the total 3 L solution into two 1.5 L containers, the system achieves higher gas velocities and more intense turbulence in each stage, thus improving the absorption of NO_x_. The cylindrical geometry with a low diameter-to-length ratio enhances bubble residence time and mixing efficiency by eliminating dead zones common in rectangular configurations.

The parametric analysis of the process revealed that operating the plasma reactor equipped with a two-stage scrubber can provide a low recovery time and low $\frac{m_{rCFs}}{E_{NOX}}$ ratios. However, the quality of the recovered fibers should be considered to establish the optimum operating conditions. Thus, the retrieved fibers were characterized by means of SEM-EDS analysis, and their mechanical properties were measured.

**Figure 7:**
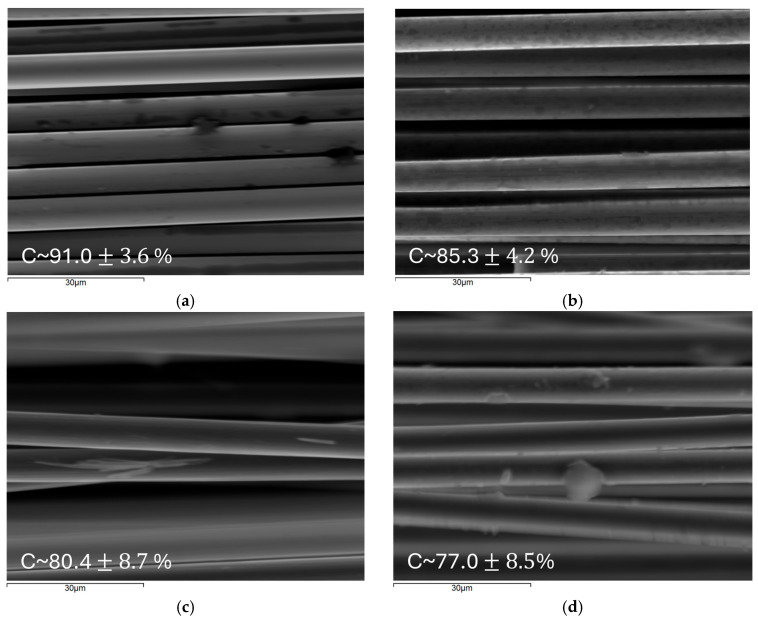
SEM-EDS images of (**a**) virgin carbon fibers (vCFs) and recovered carbon fibers (rCFs) originating from the reactor operating at 1200 W equipped with a single-stage wet scrubber with HNO_3_ concentrations of (**b**) 14 M, (**c**) 12 M, and (**d**) 10 M.

Figure 7 illustrates the SEM images and the carbon content of the virgin fibers and the recycled fibers retrieved using the scrubber-equipped reactor (1200 W, 10–14 M HNO_3_). Virgin carbon fibers (vCFs) exhibit uniform surfaces, containing a few sizing agent particles (dark particles), while maintaining the highest carbon content at 91.0 ± 3.6%. Fibers recycled with 14 M HNO_3_ achieved 85.3 ± 4.2% carbon purity with formed oxidation pits (dark “stains”) on their surface. On the other hand, fibers recycled with 12 M and 10 M HNO_3_ exhibit 80.4 ± 8.7% and 77.0 ± 8.5% carbon content, respectively, while retaining smoother surfaces with resin residual contamination (light-colored particles). This progressive reduction in purity indicates increasing levels of resin residual contamination as acid concentration decreases. The visible “stains” or darker regions observed on the surface of CFs recycled with 14 M HNO_3_ are not resin residues, as confirmed by the EDS analysis. But they represent surface oxidation layers (oxygen was the secondary compound detected by EDS) and etched surface defects introduced during the process. The literature confirms that oxidative and plasma treatments of carbon fibers create localized etch pits and surface roughening through oxidation at defect sites. At those sites, erosion does not occur uniformly but through the formation of localized etch pits, due to active surface sites with higher reactivity [58,59,60,61]. These surface modifications appear as regions of varying electron density in the SEM imaging due to selective carbon atom removal and subsequent functionalization with oxygen-containing groups, such as hydroxyl (–OH), carboxyl (–COOH), and C–O bonds [42,62,63]. The higher carbon content observed in the 14 M case demonstrates more complete matrix degradation while maintaining controlled surface oxidation. The declining surface purity at lower acid concentrations reflects both incomplete resin dissolution and excessive surface oxidation due to prolonged treatment times (16.5 h for 12 M and 20 h for 10 M). Finally, in every case, the rCFs are undamaged, with a mean diameter calculated as ~7 μm, which is equal to the diameter of the vCFs.

**Figure 8:**
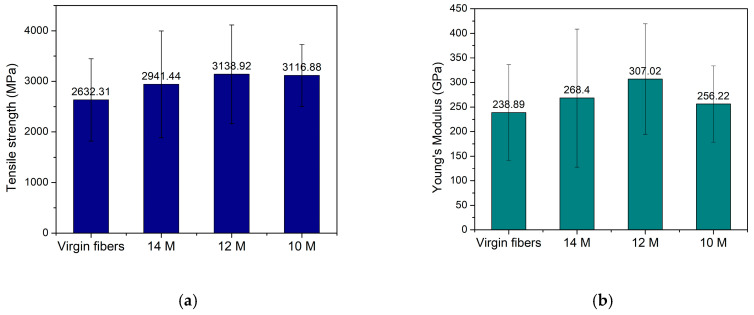
(**a**) Tensile strength and (**b**) Young modulus of the virgin carbon fibers (vCFs) and the recovered carbon fibers (rCFs) originating from the reactor operating at 1200 W equipped with a single-stage wet scrubber with HNO_3_ concentrations of 14 M, 12 M, and 10 M.

The single-fiber mechanical tests (Figure 8) reveal that plasma-assisted solvolysis produces recycled carbon fibers with retained or even slightly enhanced mechanical properties compared to virgin fibers. For tensile strength, virgin fibers achieved 2632.31 MPa, while recycled fibers with 12 M HNO_3_ reached the highest value of all tested CFs with 3138.92 MPa. Solvolysis with 10 M and 14 M HNO_3_ yielded CFs with tensile strengths of 3116.88 and 2941.44 MPa, respectively. Regarding Young’s modulus, virgin fibers exhibited 238.89 GPa, whereas recycled fibers treated with 12 M HNO_3_ again performed the best with 307.02 GPa. Moreover, CFs recycled with 10 M and 14 M HNO_3_ resulted in a Young’s modulus of 256.22 and 268.4 GPa, respectively. Single-fiber tensile tests revealed that the average tensile strength and Young’s modulus of the recycled fibers are comparable to those of the virgin fibers within the experimental uncertainty. To obtain a more robust statistical assessment, the strength data were analyzed using a two-parameter Weibull distribution. Weibull analysis further confirmed that the characteristic strength of the recycled fibers exceeds that of the virgin fibers. Hence, plasma-assisted solvolysis preserves and can even slightly enhance the mechanical integrity of the recovered fibers. Plasma-assisted solvolysis produces rCFs with retained or slightly better mechanical performance compared to the vCFs, probably due to the synergistic effects of sizing removal and controlled surface oxidation. Both plasma and nitric acid treatments oxidate the surface through controlled etching, creating oxygen-containing functional groups that heal surface defects and strengthen the carbon lattice [42,60,64,65,66]. Τhe performance at a 12 M concentration represents the ideal balance between resin removal and surface functionalization without excessive oxidative damage. Treatment with 14 M HNO_3_, despite achieving high carbon purity (85.3%), indicates that over-oxidation begins to compromise fiber integrity through excessive etch pit formation, as confirmed by the SEM imaging (Figure 7b). The 10 M condition demonstrates excellent strength retention, though incomplete resin removal suggests the creation of stress concentration sites, resulting in the lowest Young’s modulus value.

## 4. Conclusions

This work demonstrates the successful development of a process set for the pretreatment of composites, retrieval of CFs through plasma-enhanced solvolysis, cleaning of CFs, and treatment of gas and liquid wastes. Process parameters such as acid concentration, plasma power, reactor design, and wet scrubber design were tested to maximize process efficiency and minimize HNO_3_ consumption and NO_x_ emissions. Wet scrubber integration to the solvolysis system proved superior to open-air systems, reducing nitric acid consumption while enabling effective NO_x_ emission capture. The implementation of two scrubbers connected in series between them enabled almost 83% capture of NO_x_ emissions and significantly improved the environmental impact. The optimal operating conditions were identified at 14 M HNO_3_ and 1200 W, delivering complete fiber recovery in 6 h, while achieving the highest mass of recovered carbon fibers to NO_x_ emissions ratio. Recycled fibers achieved carbon purity exceeding 77% and demonstrated preserved or even slightly enhanced mechanical properties compared to those of the virgin fibers.

Compared to alternative chemical recycling methods, plasma-enhanced solvolysis still has a high negative environmental impact, mostly due to waste production [32]. But as an emerging technology, it demonstrates strong potential for further improvements in performance and environmental sustainability through continued process optimization. Moreover, while glycolysis, alcoholysis, and hydrolysis require extended treatment times (12–24 h), plasma-assisted solvolysis operates at atmospheric pressure with shorter processing times (4–6 h), lower energy demands, and higher cost-efficiency. Other chemical methods also typically degrade fiber mechanical properties, whereas plasma-assisted recycling produces fibers with retained mechanical performance.

Finally, the present results underline that the investigation of best solvolysis conditions for recycling of composites needs to be performed after considering the simultaneous impact of process parameters on CFs’ retrieval rates, CFs’ physicochemical and mechanical properties, solvent losses, and produced wastes.

## Figures and Tables

**Table 1:** Performance parameters for the single-stage and two-stage wet scrubbers during plasma-assisted solvolysis at 1200 W with 14 M nitric acid concentration.

Performance Parameters	Single-Stage Wet Scrubber	Two-Stage Wet Scrubber
HNO_3_ consumption (mol)	1.15	1.58
NO_x_ recovery (%)	48	82.67
NO_x_ emissions (mol)	0.60	0.27
$\frac{m_{rCFs}}{E_{NOX}}\;\left(\frac{g}{mol}\right)$	39.35	96.07

## Data Availability

The original contributions presented in the study are included in the article, further inquiries can be directed to the corresponding author.
